# *Plasmodium falciparum* gametocyte burden in a Tanzanian heterogeneous transmission setting

**DOI:** 10.1186/s12936-025-05270-4

**Published:** 2025-02-21

**Authors:** Charles Mulamba, Olukayode G. Odufuwa, Prisca A. Kweyamba, Linda O. Lazaro, Muhamed S. Chabo, Janeth J. Kamage, Katharina Kreppel, Ally I. Olotu, Chris L. Williams

**Affiliations:** 1 Ifakara Health Institute, 74, Bagamoyo, Tanzania; 2 Nelson Mandela African Institution of Science and Technology, Tengeru, P. O. Box, 447, Arusha, Tanzania; 3 Institute of Tropical Medicine, Antwerp, Belgium; 4 Transmission-Blocking Malaria Vaccine Group, Jenner Institute, University of Oxford, Roosevelt Drive, Headington, Oxford, OX3 7DQ UK

**Keywords:** Malaria transmission, Transmission-blocking vaccines, *Plasmodium falciparum*, Gametocytes, Tanzania

## Abstract

**Background:**

Malaria transmission depends on the presence of gametocytes in the peripheral blood of infected human hosts. Understanding malaria infectious reservoirs enables transmission-blocking interventions to target the most important hosts for the disease. This study characterized the distribution of gametocyte carriage as a baseline for the clinical evaluation of a Pfs25-based transmission-blocking vaccine candidate in Bagamoyo, Tanzania.

**Methods:**

A malaria survey was conducted in five locations from May to August 2022. A total of 467 participants—192 children (5–12 years), 65 adolescents (13–17 years) and 210 adults (18–45 years)—were enrolled. Malaria was detected using three methods: rapid diagnostic tests, light microscopy and quantitative polymerase chain reaction. The geometric mean of the gametocyte density, and weighted arithmetic mean of the gametocytes sex ratio were estimated.

**Results:**

Overall, 23.5% (110/467) of the participants tested positive for malaria parasites, with the majority of positives (> 92%) being *Plasmodium falciparum.* The overall gametocytaemia was 5.6%, with a percent positivity of 6.8% (13/192), 6.2% (4/65) and 4.3% (9/210), in children, adolescents, and adults, respectively. The geometric mean gametocyte density (gametocytes/μL) was greater in adults (124.6) than in children (71.7) and adolescents (50.5). Regression analysis revealed that gametocytes were more likely to be present among male participants than among female participants [ORa: 2.79 (95% CI: 1.19 – 6.59) p = 0.019]. The gametocyte sex ratio in children and adult gametocyte carriers was similar but greater than that in adolescents.

**Conclusion:**

The observed gametocyte densities and distribution across age groups suggest the need for malaria transmission-blocking interventions to target all populations in heterogeneous transmission settings. The implication of targeting only children may leave residual malaria transmission and reinfection from the left-out groups.

**Graphical abstract:**

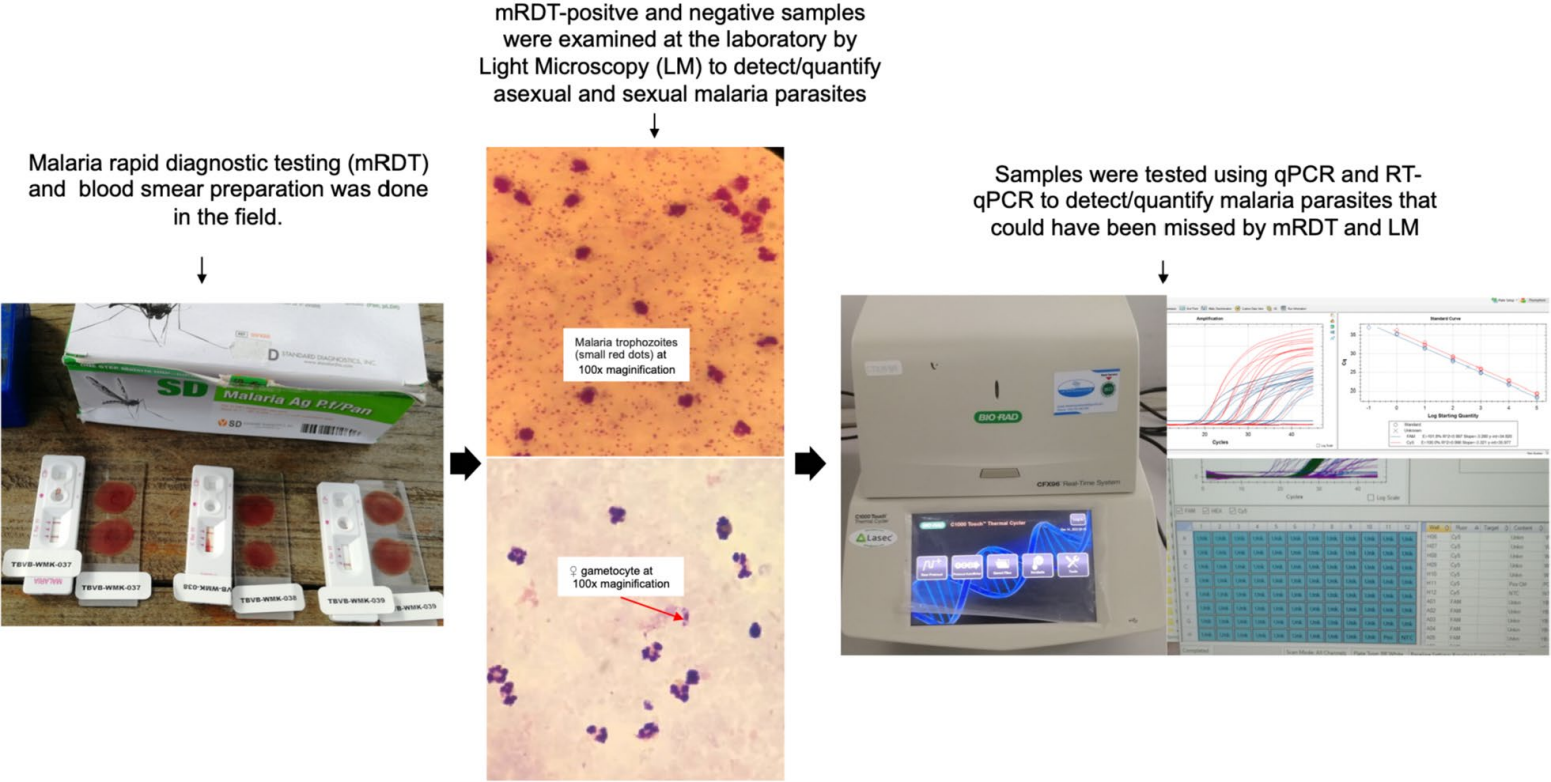

**Supplementary Information:**

The online version contains supplementary material available at 10.1186/s12936-025-05270-4.

## Background

The malaria burden is still high in the sub-Saharan Africa despite recent progress towards the Global Technical Strategy (GTS) milestones for malaria 2016–2030 [1, 2]. Tanzania is ranked among countries with high coverage and compliance to the use of core vector control tools, including insecticide treated nets and indoor residual spraying. Although the current interventions have significantly reduced the malaria burden in Tanzania [3], the country accounted for approximately 3% of all the global malaria deaths reported in 2023 [4, 5]. The malaria burden continues to persist or increasing within certain regions in the country, indicating that additional interventions are needed.

Malaria infection has a complex life cycle involving asexual and sexual stages between humans and female *Anopheles* mosquitoes [6, 7]. While clinical malaria results from parasite replication in human red blood cells (RBCs), it is the sexual forms (gametocytes), that are solely responsible for the transmission of the disease [6]. Mature (stage V) gametocytes circulate in human peripheral blood for an average of 4.6–6.5 days, and transmission occurs when at least one male and one female gametocyte are ingested by mosquitoes during a blood meal [6]. Malaria parasite transmission to mosquitoes is influenced by general parasite characteristics such as gametocyte density [8–10], concurrent asexual parasite density [11], the male to female gametocyte ratio [8, 12], and human host factors such as age and immunity [13]. Tanzania is stratified into four malaria transmission intensities based on malaria infection prevalence including very low (< 1%), low (1 < 5%), moderate (5 < 30) and high (> 30), indicating an heterogeneous transmission setting that require interventions that are not currently within the arsenal. The transmission-blocking tools are potential tools to thwart the transmission of sexual stage parasites between human and mosquitoes for an improved malaria control [14]. Efforts to develop malaria transmission-blocking tools require a thorough understanding of the human infectious reservoirs to determine the most important disease hosts to be targeted [15, 16]. School-aged children are known to harbor a greater number of gametocytes [17] and are thought to be the main target group for transmission-blocking interventions. However, the epidemiology of gametocyte carriage is not fully understood. In this study, the distribution of gametocyte carriage and natural gametocyte sex ratios are estimated, as a baseline for the clinical evaluation of a Pfs25-based transmission-blocking vaccine candidate in Bagamoyo, Tanzania.

## Methods

### Study area

**Fig. 1:**
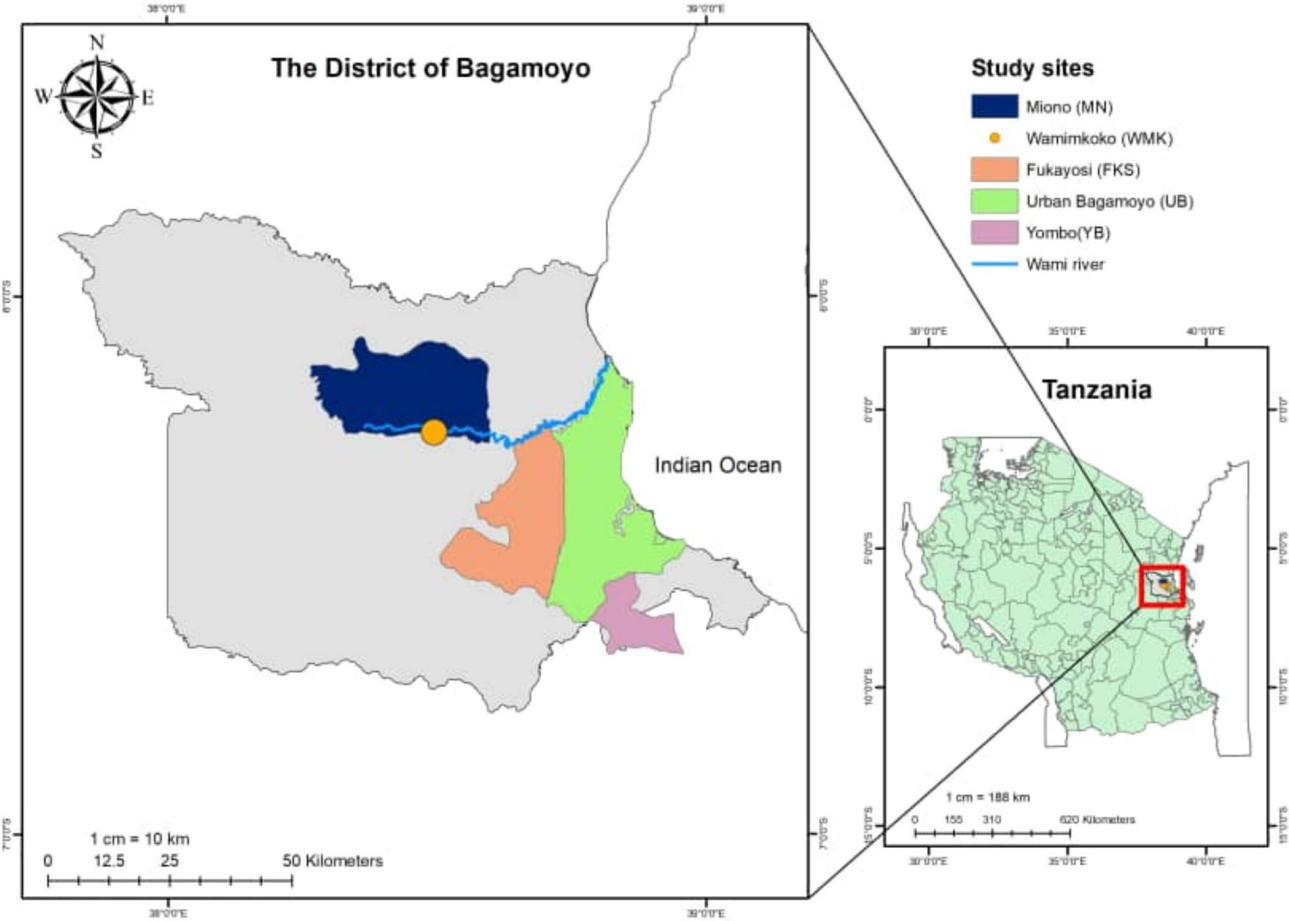
Map of the Bagamoyo district in Tanzania indicating the location of the Study sites. Participants originated from any part of each of the study sites shown on the map

The study was conducted in Bagamoyo district, which is located in Tanzania’s coastal region as shown in Fig. 1. The district represents a heterogeneous malaria transmission, with prevalence in the general population ranging between 7–39% [18, 19]. The transmission intensity is usually high during and after the long rainy season [15], which usually occurs from March to June. The majority of the reported cases are caused by *Plasmodium falciparum*, but *Plasmodium malariae* and *Plasmodium ovale* have also been reported [18, 20–22]. The five sites surveyed in Bagamoyo district have varying malaria transmission intensities: Bagamoyo township, Fukayosi, and Yombo with moderate (5 < 30% malaria infection prevalence), and Wami-Mkoko and Miono with high malaria transmission intensity (> 30% malaria infection prevalence) measured by qPCR in this study, and categorized following the guidelines of the U.S. President's Malaria Initiative (PMI) for Tanzania mainland [19]. Reported vector control practice employed in the study area are bed net with 69% coverage (households with one bed net for every two persons) and house modification with 65% of the households with screened windows and 13% with closed eaves [23].

### Study design and population

A malaria survey was conducted from May to August 2022. A total number of 467 participants, were enrolled using a simple purposively random selection from primary schools, peripheral dispensaries and community-based malaria testing camps. Participants were invited for malaria screening following mass sensitization by village health teams. All participants aged 5–45 years, without clinical symptoms for malaria, were eligible for enrollment. The participants who tested positive for malaria infection by rapid diagnostic tests (RDT) were treated with artemether-lumefantrine [24] within 24 h of diagnosis, as per the national guidelines [25].

### Blood sample collection and preservation

Blood samples were collected into 2.0-millilitre tubes containing ethylenediaminetetraacetic acid (EDTA). A total of 20 µl (µL) were used for malaria detection via RDTs and light microscopy. A volume of 200 µL was immediately preserved in 600 µL of 1 × deoxyribonucleic acid/ribonucleic acid (DNA/RNA) Shield™ (Zymo Research, Irvine, USA) for nucleic acid extraction to detect and quantify circulating parasites. Each sample was tested for malaria by RDT, light microscopy, and qPCR.

### Malaria detection by RDT and microscopy

Bioline™ Malaria Ag *P.f*/Pan (HRPII/pLDH), Standard Diagnostic, South Korea, was used for rapid malaria detection as described previously [18]. Briefly, approximately 10 μL blood was added to the sample well, followed by the addition of two drops of the standard buffer to the developer well, and left for 15 min before the results were read and recorded for each test run. Asexual parasite and gametocyte detection were performed in prepared thick smears stained for 30 min with 10% Giemsa stain and the smears were examined for the presence of *Plasmodium* parasites at 100 × magnification. In malaria parasite-positive smears, asexual parasites were counted per 200 white blood cells (WBCs) and gametocytes were counted per 500 WBCs. Malaria parasite densities were determined by assuming 8000 white blood cells per µL of blood. Smears were considered microscopy-positive for gametocytes when at least one gametocyte was observed. Smears were considered negative if no *Plasmodium* parasites were detected in 100 consecutive [26] high-power fields. Two microscopists examined the slides independently with a difference of 4.5% discordant slides between the two microscopists. A third microscopist re-examined the discordant smears to confirm the results.

### Malaria detection and quantification by qPCR

Parasitaemia was determined by quantitative polymerase chain reaction (qPCR) performed on the blood samples preserved in DNA/RNA Shield^™^ [27, 28]. Genomic DNA was extracted from *Plasmodium* positive blood samples using a Quick-DNA Miniprep Plus Kit (Zymo Research, USA), and eluted in 50 µL of elution buffer. The qPCR targeting the Pan-Plasmodium 18S rRNA and *P. falciparum*-specific varATS sequences, was performed. This approach enhances both the specificity and sensitivity for detecting *P. falciparum* as well as non-*falciparum Plasmodium* species. The single qPCR reaction contained 2μL of parasite DNA and 8 µL of reaction master mix containing 1 × Luna Universal Probe qPCR Master Mix (New England Biolabs, USA). The samples were run in duplicate along with negative controls and *P. falciparum* NF54 DNA as a positive control. To investigate other *Plasmodium* species infections in the study sites, all *Plasmodium*-positive samples were further analysed by including controls for *Plasmodium malariae*, and *Plasmodium ovale* in the assay [29]. qPCR was performed on a CFX96 real-time qPCR thermocycler (Bio-Rad, Singapore), and the results were analysed with CFX Manager Software (v2.2). The following thermal profile was used: polymerase activation at 95 °C for 60 s (s), 45 cycles of denaturation at 95 °C for 15 s, and annealing and elongation for 45 s at 57 °C. The number of parasites per µL of blood was calculated against the World Health Organization (WO) international standard for *P. falciparum* DNA quantification techniques [27]. The standard was serially diluted from 100,000 parasites/µL—0.0001 parasites/µL—and run in triplicate [29].

The primers and probes used in Schindler et al., 2019 [29], were used as described in Hofer et al*.*, 2023 [18]. Sample analysis was performed using the same qPCR system, reaction volumes, and materials as described above, except that the cycling conditions used for the assay were as follows: activation for 5 min at 95 °C; 45 cycles of denaturation for 15 s at 95 °C and annealing and elongation for 45 s at 57 °C.

### Gametocyte detection by RT‒qPCR

Ribonucleic acid (RNA) was extracted from all qPCR-positive samples, using the Quick-RNA™ MiniPrep Plus kit (Zymo Research). The protocol was adapted according to the manufacturer’s instructions as previously described [18]. *Plasmodium falciparum* gametocytes were detected and quantified using a multiplex qRT‒PCR assay described previously by Meerstein‑Kessel et al., [30]. The assay combined two independent *Plasmodium* targets: the female gametocyte-specific marker (CCp4) and the male gametocyte-specific marker (*Pf*MGET), as detailed in Additional file 1. Reverse transcription, amplification, and qPCR measurements were performed using the same instrument as above with the following thermal profile: 15 min at 55 °C; 60 s at 95 °C; 40 cycles of 10 s 95 °C and 60 s at 60 °C. Each reaction contained 4 µL RNA and 10 µL reaction master mix containing Luna Universal 1 × One-Step qRT‒PCR Kit (New England Biolabs, USA). The standard curves for gametocyte quantification were prepared using RNA standards (obtained from the Department of Medical Microbiology, Radboud University Medical Center, Nijmegen, The Netherlands) for sex-sorted gametocytes [26, 28]. A tenfold dilution series in the range of 10^6^/mL to 10^1^/mL was made for each standard and used for standard curve preparation. The reaction efficiencies for *Pf*MGET and CCp4 were 95.7% and 80.0%, respectively, as shown in Additional file 2.

### Statistical analysis

The number of participants enrolled was estimated using the formula for prevalence studies [31] as follows: $\text{n}=\frac{{\text{Z}}^{2}\text{P }\left(1 -\text{ P}\right)}{{\text{d}}^{2}}$, where n is the sample size, Z is the standard normal variate of 1.96 at a confidence level of 95%, and d the precision of 0.05 precision. P is the previous malaria prevalence (10%) in Bagamoyo district at the 95% confidence interval. A minimum of 139 participants was required for the study.

The data were recorded in Excel (Microsoft, 2016) and analysed using Stata version 16 [32] and GraphPad Prism version 10 (GraphPad Software Inc., California, USA). Descriptive analyses were conducted on the demographic characteristics [age groups (5–12, 13–17, and 18–45 years), sex (male and female), and location [moderate (5 < 30% malaria infection prevalence) and high (> 30% malaria infection prevalence) transmission] of the participants detected by qPCR using proportions and their respective 95% confidence intervals (CIs).

The geometric mean (GM) of the gametocyte density and the weighted arithmetic mean of the gametocyte sex ratio estimated by dividing male gametocytes by total number of gametocytes are presented. A t-tests were performed on the natural log of gametocyte density in relation to the demographic characteristics. Binary logistic regression was used to estimate the associations between the presence of gametocytes and the demographic characteristics of the participants. Likelihood ratio tests were also performed to assess the influence of each of the variables in the multivariate model.

## Results

### Study participants

A total of 467 participants, including 41% children (5–12 years), 14% adolescents (13–17 years), and 45% adults (18–45 years), were recruited, as shown in Table 1. The median age (years) in each age group was 8, 14, and 30, for children, adolescents, and adults, respectively. The proportion of female participants was 54%. From the overall 110 participants that tested positive for malaria parasite by qPCR, the prevalence in Bagamoyo township was 10%, Fukayosi was 14% and Yombo was 6%, and were all categorized as low malaria transmission, and those categorized as a high malaria transmission were Miono with 32% and Wami-Mkoko with 39% malaria infection prevalence. Participant recruitment among these villages varied slightly between low (44%) and high (56%) malaria transmission sites.

**Table 1:** Demographic characteristics of study participants

Demographic variables (N = 467)	n (%)
Age-group in years	
Children (5–12)	192 (41.1)
Adolescent (13–17)	65 (13.9)
Adult (18–45)	210 (45.0)
Sex	
Female	252 (54.0)
Male	215 (46.0)
Study site	
Moderate Transmission (5 < 30% prevalence)	205 (43.9)
High Transmission (> 30% prevalence)	262 (56.1)

### Asexual parasite prevalence RDT, microscopy, and qPCR methods

**Fig. 2:**
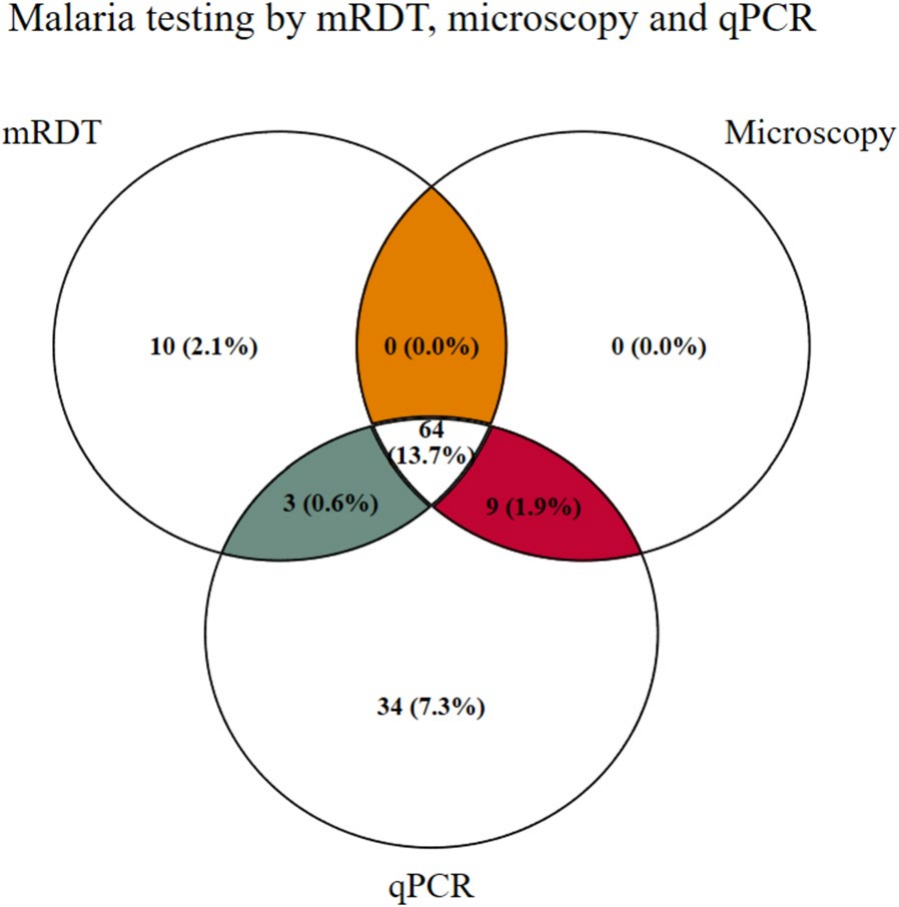
Concordance in malaria testing methods for positive samples (n = 110)

All three malaria diagnostic tools detected higher asexual parasite positivity rates in children and adolescents, than in adults (Table 2). With respect to all groups combined, qPCR detected the greatest number of infections (n = 110), with a mean asexual parasite density of 391.8 copies/μL. Microscopy detected 73 infections, with a mean asexual parasite density of 1170 copies/μL. Only 67 true asexual positives could be detected by the RDT. There were 10 false malaria-positive infections detected by RDT as shown in Fig. 2.

**Table 2:** Asexual parasite prevalence across age groups by test methods: RDT, microscopy and qPCR

Testing method	Children		Adolescents		Adults	
	n	% (95% CI)	n	% (95% CI)	n	% (95% CI)
mRDT						
Asexual	41	21.4 (16.1–27.7)	17	26.2 (16.9–38.1)	19	9.0 (5.8–13.8)
LM						
Asexual	43	22.4 (17.0 – 28.9)	13	20.0 (12.0 – 31.5)	17	8.0 (5.1–12.6)
Sexual	8	4.2 (2.1 – 8.1)	3	4.6 (1.5 – 13.4)	6	2.9 (1.3–6.2)
qPCR						
Asexual	52	27.1 (21.3 – 33.8)	19	29.2 (19.5 – 41.4)	39	18.6 (13.9–24.4)
Sexual	13	6.8 (4.0 – 11.3)	4	6.2 (2.3 – 15.3)	9	4.3 (2.2–8.0)

Using sensitive qPCR as the reference standard, the majority of the malaria-positive participants [93% (102/110)] were infected with *P. falciparum*, as detailed in Table 3. One participant had *P. malariae* infection only, and the remaining malaria positive participants (n = 7) had mixed infections of *P. malariae* and *P. falciparum*. The geometric mean asexual parasite density in the children (746.1 copies/μL) was four-fold greater than that in the adolescents (192.6 copies/μL), and threefold greater than that in the adults (234.6 copies/μL). The asexual parasite positivity in male participants was higher compared to their female counterparts, but a similar mean asexual parasite density was observed in both sexes (Table 3).

**Table 3:** Malaria parasite positivity by qPCR in relation to participants’ demographic characteristics

Demographics	Asexual parasites (N = 467)			Detected parasite species distribution					
				*P. falciparum* (*Pf*)		*P. malariae* [45]		*Pf* & *Pm*	
	n^t^	%^t^ (95% C I)	G. Mean parasite density (95% CI)	n^f^	%^e^ (95% C I)	n^m^	%^m^ (95% C I)	n^d^	%^h^ (95% C I)
Overall	110	23.6 (19.9–27.6)	391.8 (232.1–661.4)	102	92.7 (86.0 – 96.3)	01	0.9 (0.1 – 6.3)	07	6.4 (3.0 – 12.8)
Age-group									
Children	52	27.1 (21.3–33.8)	746.1 (330.9–1682.3)	48	92.3 (81.1 – 97.1)	01	1.9 (0.3 – 12.7)	03	5.8 (1.8 – 16.6)
Adolescent	19	29.2 (19.5 – 41.4)	192.6 (79.1 – 469.2)	17	89.5 (65.9 – 97.4)	00	00	02	10.5 (2.6 – 34.1)
Adult	39	18.6 (13.9 – 24.4)	234.6 (94.5 – 582.6)	37	94.9 (81.4 – 98.7)	00	00	02	5.1 (1.3 – 18.6)
Sex									
Female	52	20.6 (16.1 – 26.1)	386.8 (161.7 – 925.2)	47	90.4 (78.7 – 96.0)	01	1.9 (0.3 – 12.7)	04	7.7 (2.9 – 18.9)
Male	58	27.0 (21.5 – 33.3)	396.3 (209.2 – 750.8)	55	94.8 (85.0 – 98.3)	00	00	03	5.2 (1.7 – 15.0)
Location (Transmission intensity)									
Low	20	9.8 (6.4 – 14.7)	1,534.1 (312.0 – 7,543.5)	20	100	0	0	0	0
High	90	34.4 (28.8 – 40.3)	289.3 (170.2 – 491.8)	82	91.1 (83.1 – 95.5)	1	1.1 (0.2 – 7.6)	7	7.8 (4.7 – 15.5)

N = 467 participants tested for malaria parasites

n^t^ = total number of participants who tested positive for malaria

%t = n^t^/N*100

n^f^ = number of *Plasmodium falciparum* infections

n^m^ = number of *Plasmodium malariae* infections

n^d^ = number of mixed infections with *Plasmodium falciparum* and *Plasmodium malariae*

%^f^ = n^f^/n^t^*100

%^m^ = n^m^/n^t^*100

%^d^ = n^d^/n^t^*10

### *Plasmodium falciparum* gametocyte prevalence by qRT‒PCR

**Fig. 3:**
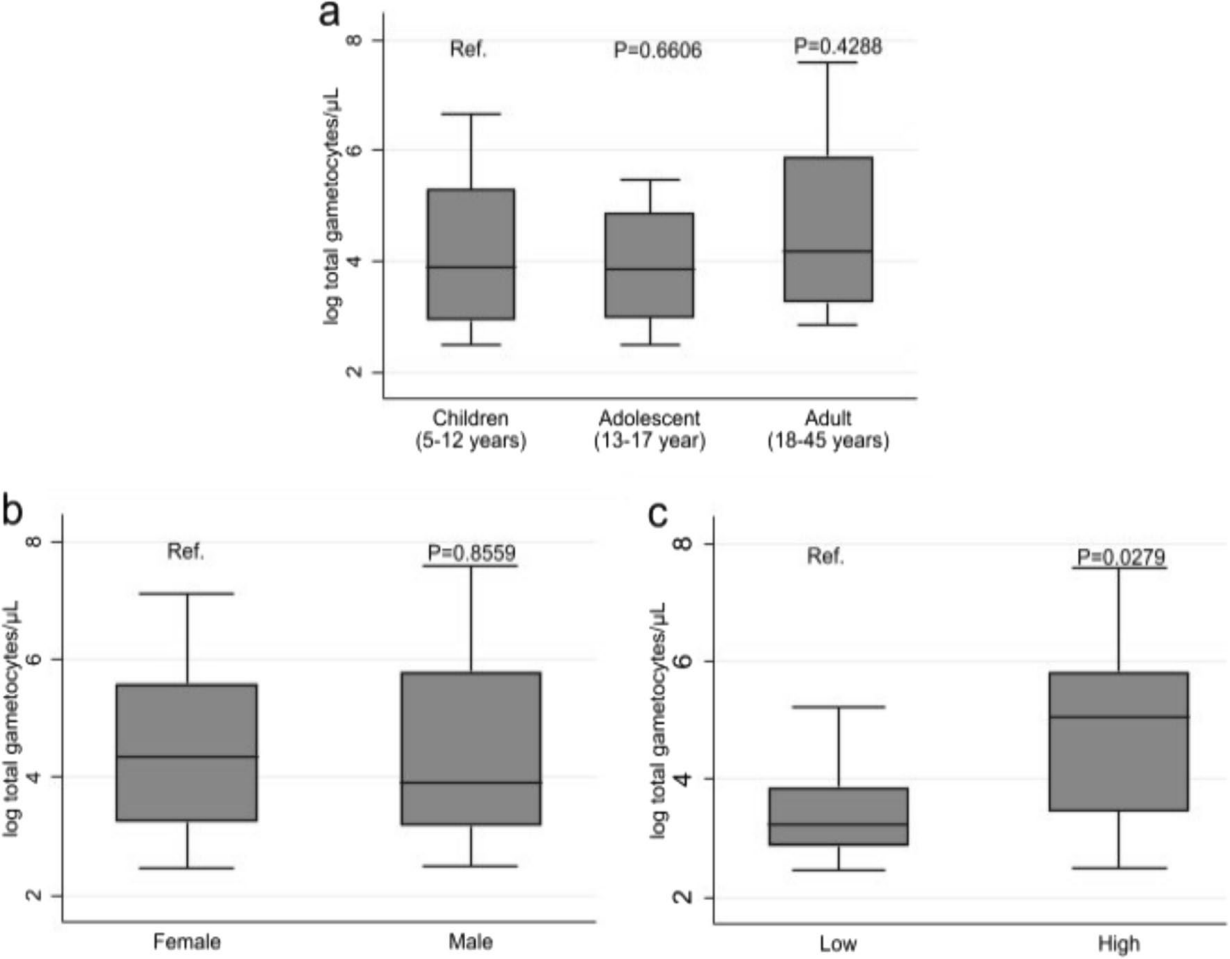
The distribution of Ct-based estimated gametocyte densities Gametocyte density by participants’ age (**a**), sex (**b**) and location (**c**).

The overall gametocyte prevalence according to qRT‒PCR was 5.6% (26/467), and the overall gametocyte sex ratio (proportion of male gametocytes), was 0.18 (Table 4). Approximately 24% (26/110) of the malaria-positive participants had gametocytes. Gametocyte prevalence was lower among adults than other age groups, and higher among males than among their female counterparts, as well as among those living in high transmission areas compared to participants from low transmission areas (Table 4 and Fig. 3). However, the geometric mean (GM) gametocyte density was greater in adults (124.6 gametocytes/μL) than in children (71.7) and adolescents (50.5). A similar pattern of greater GM density was also observed among those living in the high transmission location (126.0) than among those the low transmission areas (31.5). The gametocyte sex ratio in children and adult gametocyte carriers was similar but greater than that was observed in adolescents as indicated in Table 4.

**Table 4:** Gametocyte positivity by RT-qPCR in relation to participants’ demographic characteristics

Demographics	n^g^	%^g^ (95% CI)	G. Mean gametocyte density (95% CI)	Weighted A. Mean gametocyte sex ratio (95% CI)
Overall	26	5.6 (3.8 – 8.1)	82.2 (44.6 –151.6)	0.18 (0.15 – 0.21)
Age-group				
Children	13	6.8 (4.0 – 11.3)	71.7 (30.8 – 167.0)	0.20 (0.15 – 0.25)
Adolescent	04	6.2 (2.3 – 15.3)	50.5 (6.8 – 376.7)	0.08 (0 – 0.25)
Adult	09	4.3 (2.2 – 8.0)	124.6 (31.0 – 500.9)	0.18 (0.14 – 0.22)
Sex				
Female	08	3.2 (1.6 – 6.2)	89.4 (23.9 – 334.1)	0.14 (0.09 – 0.19)
Male	18	8.4 (5.3 – 12.9)	79.3 (37.0 – 169.6)	0.19 (0.15 – 0.24)
Location (transmission intensity)				
Low	08	3.9 (2.0 – 7.6)	31.5 (15.0 – 66.2)	0.20 (0.16 – 0.24)
High	18	6.9 (4.4 – 10.7)	126.0 (58.2 – 273.0)	0.17 (0.13 – 0.22)

N = 467 participants tested for gametocytes

n^g^ = number of participants who tested positive for gametocytes

%^g^ = n^g^/N*100

sex ratio = number of male gametocytes/total gametocyte count

Approximately 35% (9/26) of gametocyte carriers identified by RT‒qPCR, were sub-microscopic and could not be detected by microscopy. Of the 9 sub-microscopic gametocyte carriers, 3 were adults, 5 were children and one adolescent. Additional file 3 shows the results of malaria detection by RDT and microscopy.

Binary logistic regression was performed to assess whether age, sex or location influenced gametocytaemia, and the results indicated that there was no association between the gametocytaemia and age (P-value = 0.7107), which was consistent with the lack of a significant difference in the log of gametocyte density among the age groups (Table 4 and Fig. 3a). Although it was shown that gametocytes were more likely to be present among the male participants than among female participants [ORa: 2.79 (95% CI 1.19 – 6.59) p = 0.019] (Table 4), the GM of gametocyte density did not significantly differ between male and female participants (Fig. 3b). The GM gametocyte density was significantly greater among the participants in the high transmission areas than those in low transmission areas (Fig. 3c), however, there was no significant difference between location and gametocyte prevalence (Table 5).

**Table 5:** Association of gametocyte carriage with age, sex, and study site

	Unadjusted		Adjusted		LR test
	OR (95% CI)	P-value	OR (95% CI)	P-value	P-value
Age-group					
Adult	1.00		1.00		0.7107
Children	1.62 (0.68 – 3.88)	0.278	1.45 (0.59 – 3.55)	0.412	
Adolescent	1.46 (0.44 – 4.92)	0.537	1.19 (0.35 – 4.08)	0.779	
Sex					
Female	1.00		1.00		0.0144*
Male	2.79 (1.19 – 6.54)	0.019*	2.81 (1.19 – 6.65)	0.019	
Location (by transmission intensity)					
Low	1.00		1.00		0.1994
High	1.82 (0.77 – 4.27)	0.171	1.72 (0.73 – 4.17)	0.211	

Sex, age group and location are included in the adjusted model

^*^Statistically significant

## Discussion

A better understanding of the infectious reservoir of *P. falciparum* will enhance the development and evaluation of new tools that can interrupt malaria transmission. Here, the malaria parasite prevalence and density in school-age children, adolescents, and adults from the Bagamoyo district in Tanzania were determined. In line with common practices in many African countries, malaria rapid diagnostic tests (RDTs) and microscopy were utilized to detect malaria infections in this study. Additionally, sensitive qPCR assays were employed to ensure the detection of asymptomatic infections that may be missed by RDT and microscopy.

A large proportion of gametocyte carriers in this study were adults, indicating a shift in the age distribution of gametocyte carriage. This shift could be attributed to intensive efforts to reduce and prevent malaria in children than in adults in recent years [23, 33].

As a tradition, men and young boys in this study area are more engaged in malaria-risk activities, such as fishing at night, animal grazing among others. This could explain why a higher gametocyte prevalence was observed in male participants compared with their female counterparts.

There have been previous reports of relatively high gametocyte prevalence detected by qPCR in school-age children in Bagamoyo district than what was observed in this study using qPCR; one study in Kiwangwa village reported a gametocyte prevalence of 14% [34], and another study in Buma and Yombo reported a gametocyte prevalence of 18% [18]. Given the difference in study villages, a direct comparison between studies may not be appropriate. Nonetheless, the difference between the gametocyte prevalence in this study and previous reports could be reflective of heterogeneity in malaria transmission and carriage rates in the population.

It was observed that microscopy missed more than one-third (33.6%) of infections, whereas RDT missed nearly 40% of the qPCR-positive infections. These findings corroborate previous studies in Tanzania [28], Nigeria [35], and Ghana [36], where significant proportions of qPCR-positive infections were missed by microscopy and RDT. The missed infections recorded by microscopy and RDT in the current study could be attributed to submicroscopic parasites, which tend to persist for several months without any symptoms. Furthermore, the observed RDT false negatives may have been a result of HRP-2 gene deletion, which is prevalent in Tanzania [37]. The missed infections are of great concern since failure to identify malaria carriers may lead to continued transmission and an increase in the malaria burden, particularly in low-transmission areas. The observed false positives by RDT may be a result of the persistence of the HRP2 antigen after successful anti-malarial treatment. HRP2 positivity is more common when treatment is done with artemisinin combination therapy and may last for 2–20 days post-treatment [38].

Approximately 24% of malaria-positive participants in this study were found to harbour *P. falciparum* gametocytes, underscoring the prevalence of these sexual-stage parasites in a predominantly asymptomatic population where malaria infections are often sub-microscopic [18, 24, 34]. These results align with findings from several studies conducted in other malaria-endemic areas [39–42], which have reported similar age distributions.

The overall gametocyte sex ratio in the current study is similar to what has been observed in other endemic areas [8]. The gametocyte sex ratio in adult gametocyte carriers in this study is an indication that adults could also be playing a significant role in malaria transmission. The gametocyte sex ratio, which is; the proportion of male gametocytes, is a useful predictor of the malaria infectiousness of human host and their potential to transmit malaria parasites [43]. Although the likelihood of mosquito infection largely depends on gametocyte density, parasite fertilization in the mosquito requires sufficient numbers of male and female gametocytes to be present in the mosquito blood meal [44]. Quantifying both male and female gametocytes in human hosts allows a better prediction of infectiousness than measurement of the total gametocytes or the more abundant female gametocytes [8].

The gametocytes in one third of the gametocyte carriers were sub microscopic, highlighting the need for molecular testing in malaria surveillance and evaluation of interventions [24, 39]. Light microscopy is the gold standard for parasite detection but the use of highly sensitive molecular diagnostic tools is important in heterogeneous endemic settings, and where sub-microscopic infections are common. A recent study in Bagamoyo correlated gametocyte presence with infectiousness in mosquitoes, indicating that 95% of all oocyst-infected mosquitoes after direct membrane feeding assays were due to sub-microscopic malaria infections in school-age children [18].

The strength of this study lies in the fact that both asexual and gametocyte positivity was measured in children, adolescents and adults. Previous studies conducted in Tanzania focused on either child below 10 years and/or adolescents, who were recruited from schools and health facilities [18, 34]. The assessment of asexual and gametocyte prevalence provides an indication for the magnitude of the infection and the potential infective reservoir in the study area. This information is essential for effective malaria control in the study area and Tanzania at large. In addition, three testing methods were used for parasite detection in this study, which reaffirms the results.

However, this study encountered some limitations; despite recruiting more than the total number of participants estimated by power calculation, the number of adolescents recruited was limited. In addition, participants were recruited from only five locations, which were easily accessible. This may have restricted the study area and sample size. Lastly, this study was unable to determine the infectiousness to mosquitoes across the different age groups to ascertain actual infectiousness by age.

While further prospective studies are warranted to fully elucidate the age-specific contribution to mosquito infection, particularly given a higher gametocyte density observed in adults, these findings hold important implications for community-level malaria control efforts. Notably, adults, like school-age children, constitute a substantial proportion of gametocyte carriers in the study area, suggesting that interventions focused solely on children may not suffice to effectively interrupt malaria transmission.

## Conclusion

Malaria prevalence was higher among children and adolescents compared to adults but *Plasmodium falciparum* gametocytaemia appeared to be distributed evenly across different age groups. Hence, when developing and evaluating transmission-blocking interventions, it is crucial to consider the demographic characteristics of the target population. Solely focusing on children may lead to residual malaria transmission and potential re-infection from other left-out age groups.

## Supplementary Information

Additional file 1. Primers and probes used for gametocyte detection and quantification by RT‒qPCR.

Additional file 2. RT‒qPCR reaction efficiency for *Pf*MGET and CCp4 markers used for the detection and quantification of male and female gametocytes, respectively, in the malaria survey participants.

Additional file 3. Malaria parasite positivity RDT and microscopy.

Additional file 4. Complete data set of the current study.

## Data Availability

All data generated or analysed during this study are included as Additional file 4.

## Abbreviations

ALU: Artemether–lumefantrine
DNA: Deoxyribonucleic acid
CI: Confidence Interval
HRP-2: Histidine-rich protein 2
LM: Light microscopy
RDT: Rapid diagnostic test
NIBSC: National institute for biological standards and control
NIMR: National institute of medical research
pLDH: Parasite lactate dehydrogenase
qPCR: Quantitative polymerase chain reaction
RBC: Red blood cell
RNA: Ribonucleic acid
RT- qPCR: Reverse transcription-quantitative polymerase chain reaction
WBC: White blood cell
WHO: World Health Organization

## References

[1] WHO. World malaria report. 20 years of global progress and challenges. Geneva: World Health Organization; 2020. p. 2020.

[2] WHO. World malaria report 2021. Geneva: World Health Organization; 2021.

[3] PROGRAMME NMC. National Malaria Strategic Plan 2014–2020: Abridged Version. In: Programme NMC, editor. Dar es salaam The Government of Tanzania; 2014.

[4] WHO. World Malaria report. Geneva: World Health Organization; 2023.

[5] WHO. World malaria report 2022. Geneva: World Health Organization; 2022.

[6] Reuling IJ, Van De Schans LA, Coffeng LE, Lanke K, Meerstein-Kessel L, Graumans W, et al. A randomized feasibility trial comparing four antimalarial drug regimens to induce *Plasmodium falciparum* gametocytemia in the controlled human malaria infection model. Elife. 2018;7: e31549. 29482720 10.7554/eLife.31549 PMC5828662

[7] Aguilar R, Magallon-Tejada A, Achtman AH, Moraleda C, Joice R, Cisteró P, et al. Molecular evidence for the localization of *Plasmodium falciparum* immature gametocytes in bone marrow. Blood. 2014;123:959–66. 24335496 10.1182/blood-2013-08-520767 PMC4067503

[8] Bradley J, Stone W, Da DF, Morlais I, Dicko A, Cohuet A, et al. Predicting the likelihood and intensity of mosquito infection from sex specific *Plasmodium falciparum* gametocyte density. Elife. 2018;7: e34463. 29848446 10.7554/eLife.34463 PMC6013255

[9] Da DF, Churcher TS, Yerbanga RS, Yaméogo B, Sangaré I, Ouedraogo JB, et al. Experimental study of the relationship between *Plasmodium* gametocyte density and infection success in mosquitoes; implications for the evaluation of malaria transmission-reducing interventions. Exp Parasitol. 2015;149:74–83. 25541384 10.1016/j.exppara.2014.12.010

[10] Collins KA, Wang CY, Adams M, Mitchell H, Rampton M, Elliott S, et al. A controlled human malaria infection model enabling evaluation of transmission-blocking interventions. J Clin Invest. 2018;128:1551–62. 29389671 10.1172/JCI98012 PMC5873858

[11] Lin Ouédraogo A, Gonçalves BP, Gnémé A, Wenger EA, Guelbeogo MW, Ouédraogo A, et al. Dynamics of the human infectious reservoir for malaria determined by mosquito feeding assays and ultrasensitive malaria diagnosis in Burkina Faso. J Infect Dis. 2016;213:90–9. 26142435 10.1093/infdis/jiv370

[12] Paul RE, Brey PT, Robert V. *Plasmodium* sex determination and transmission to mosquitoes. Trends Parasitol. 2002;18:32–8. 11850012 10.1016/s1471-4922(01)02122-5

[13] Smith RC, Vega-Rodríguez J, Jacobs-Lorena M. The *Plasmodium* bottleneck: malaria parasite losses in the mosquito vector. Mem Instit Oswaldo Cruz. 2014;109:644–61. 10.1590/0074-0276130597 PMC4156458 25185005

[14] Coutinho-Abreu IV, Ramalho-Ortigao M. Transmission blocking vaccines to control insect-borne diseases: a review. Mem Instit Oswaldo Cruz. 2010;105:1–12. 10.1590/s0074-02762010000100001 20209323

[15] Bousema T, Roeffen W, Meijerink H, Mwerinde H, Mwakalinga S, van Gemert G-J, et al. The dynamics of naturally acquired immune responses to *Plasmodium falciparum* sexual stage antigens Pfs230 & Pfs48/45 in a low endemic area in Tanzania. PLoS ONE. 2010;5: e14114. 21124765 10.1371/journal.pone.0014114 PMC2993929

[16] Bousema J, Drakeley C, Kihonda J, Hendriks J, Akim N, Roeffen W, et al. A longitudinal study of immune responses to *Plasmodium falciparum* sexual stage antigens in Tanzanian adults. Parasite Immunol. 2007;29:309–17. 17518949 10.1111/j.1365-3024.2007.00948.x

[17] Churcher TS, Bousema T, Walker M, Drakeley C, Schneider P, Ouédraogo AL, et al. Predicting mosquito infection from *Plasmodium falciparum* gametocyte density and estimating the reservoir of infection. Elife. 2013;2: e00626. 23705071 10.7554/eLife.00626 PMC3660740

[18] Hofer LM, Kweyamba PA, Sayi RM, Chabo MS, Maitra SL, Moore SJ, et al. Malaria rapid diagnostic tests reliably detect asymptomatic *Plasmodium falciparum* infections in school-aged children that are infectious to mosquitoes. Parasit Vectors. 2023;16:217. 37391770 10.1186/s13071-023-05761-w PMC10314504

[19] PMI. Tanzania (Mainland) Malaria Profile. 2023.

[20] Mwangonela Z. Field evaluation of a novel one step malaria pf and pf/pv rapid diagnostic tests in Pwani region, Tanzania: NM-AIST; 2023. 10.1186/s42269-023-00992-4 PMC9904258 36776799

[21] Tarimo BB, Nyasembe VO, Ngasala B, Basham C, Rutagi IJ, Muller M, et al. Seasonality and transmissibility of *Plasmodium ovale* in Bagamoyo District. Tanzania Parasit Vectors. 2022;15:56. 35164867 10.1186/s13071-022-05181-2 PMC8842944

[22] Popkin-Hall ZR, Seth MD, Madebe RA, Budodo R, Bakari C, Francis F, et al. Malaria species positivity rates among symptomatic individuals across regions of differing transmission intensities in Mainland Tanzania. medRxiv. 2023;2:141. 10.1093/infdis/jiad522 PMC11011190 37992117

[23] Odufuwa OG, Ross A, Mlacha YP, Juma O, Mmbaga S, Msellemu D, et al. Household factors associated with access to insecticide-treated nets and house modification in Bagamoyo and Ulanga districts, Tanzania. Malar J. 2020;19:220. 32576180 10.1186/s12936-020-03303-8 PMC7313165

[24] Shekalaghe SA, Teun Bousema J, Kunei KK, Lushino P, Masokoto A, Wolters LR, et al. Submicroscopic *Plasmodium falciparum* gametocyte carriage is common in an area of low and seasonal transmission in Tanzania. Trop Med Int Health. 2007;12:547–53. 17445146 10.1111/j.1365-3156.2007.01821.x

[25] Ministry of Health and Social Welfare. National guidelines for diagnosis and treatment of malaria. National Malaria Control Programme, Dar es Salaam, Tanzania, 2011.

[26] Conway DJ, Machado RL, Singh B, Dessert P, Mikes ZS, Povoa MM, et al. Extreme geographical fixation of variation in the *Plasmodium falciparum* gamete surface protein gene Pfs48/45 compared with microsatellite loci. Mol Biochem Parasitol. 2001;115:145–56. 11420101 10.1016/s0166-6851(01)00278-x

[27] Kamau E, Alemayehu S, Feghali KC, Saunders D, Ockenhouse CF. Multiplex qPCR for detection and absolute quantification of malaria. PLoS ONE. 2013;8: e71539. 24009663 10.1371/journal.pone.0071539 PMC3756973

[28] Hofmann N, Mwingira F, Shekalaghe S, Robinson LJ, Mueller I, Felger I. Ultra-sensitive detection of *Plasmodium falciparum* by amplification of multi-copy subtelomeric targets. PLoS Med. 2015;12: e1001788. 25734259 10.1371/journal.pmed.1001788 PMC4348198

[29] Schindler T, Robaina T, Sax J, Bieri JR, Mpina M, Gondwe L, et al. Molecular monitoring of the diversity of human pathogenic malaria species in blood donations on Bioko Island Equatorial Guinea. Malar J. 2019;18:9. 30646918 10.1186/s12936-019-2639-8 PMC6332537

[30] Meerstein-Kessel L, Andolina C, Carrio E, Mahamar A, Sawa P, Diawara H, et al. A multiplex assay for the sensitive detection and quantification of male and female *Plasmodium falciparum* gametocytes. Malar J. 2018;17:441. 30497508 10.1186/s12936-018-2584-y PMC6267050

[31] Charan J, Biswas T. How to calculate sample size for different study designs in medical research? Indian J Psychol Med. 2013;35:121–6. 24049221 10.4103/0253-7176.116232 PMC3775042

[32] StataCorp. Stata Statistical Software: Release 16. College Station, TX: StataCorp LLC. 2019.

[33] Winch PJ, Makemba AM, Kamazima SR, Lurie M, Lwihula GK, Premji Z, Minjas JN, Shiff CJ. Local terminology for febrile illnesses in Bagamoyo District, Tanzania and its impact on the design of a community-based malaria control programme. Soc Sci Med. 1996;42(7):1057–67. 8730911 10.1016/0277-9536(95)00293-6

[34] Sumari D, Mwingira F, Selemani M, Mugasa J, Mugittu K, Gwakisa P. Malaria prevalence in asymptomatic and symptomatic children in Kiwangwa, Bagamoyo district, Tanzania. Malar J. 2017;16:222. 28545457 10.1186/s12936-017-1870-4 PMC5445421

[35] Umunnakwe FA, Idowu ET, Ajibaye O, Etoketim B, Akindele S, Shokunbi AO, et al. High cases of submicroscopic *Plasmodium falciparum* infections in a suburban population of Lagos. Nigeria Malar J. 2019;18:433. 31856852 10.1186/s12936-019-3073-7 PMC6924037

[36] Opoku Afriyie S, Addison TK, Gebre Y, Mutala A-H, Antwi KB, Abbas DA, et al. Accuracy of diagnosis among clinical malaria patients: comparing microscopy, RDT and a highly sensitive quantitative PCR looking at the implications for submicroscopic infections. Malar J. 2023;22:76. 36870966 10.1186/s12936-023-04506-5 PMC9985253

[37] Mwangonela ZE, Ye Y, Rachel Q, Msuya HM, Mwamlima TG, Mswata SS, et al. Field evaluation of the novel One Step Malaria Pf and Pf/Pv rapid diagnostic tests and the proportion of HRP-2 gene deletion identified on samples collected in the Pwani region, Tanzania. Bull Nat Res Cent. 2023;47:17. 10.1186/s42269-023-00992-4 PMC9904258 36776799

[38] Oulton T, Mahamar A, Sanogo K, Diallo M, Youssouf A, Niambele SM, et al. Persistence of *Plasmodium falciparum* HRP-2 antigenaemia after artemisinin combination therapy is not associated with gametocytes. Malar J. 2022;21:372. 36474274 10.1186/s12936-022-04387-0 PMC9724264

[39] Coalson JE, Walldorf JA, Cohee LM, Ismail MD, Mathanga D, Cordy RJ, et al. High prevalence of *Plasmodium falciparum* gametocyte infections in school-age children using molecular detection: patterns and predictors of risk from a cross-sectional study in southern Malawi. Malar J. 2016;15:527. 27809907 10.1186/s12936-016-1587-9 PMC5096312

[40] Koepfli C, Robinson LJ, Rarau P, Salib M, Sambale N, Wampfler R, et al. Blood-stage parasitaemia and age determine plasmodium falciparum and P vivax gametocytaemia in Papua New Guinea. PLoS One. 2015. 10.1371/journal.pone.0126747. 25996916 10.1371/journal.pone.0126747 PMC4440770

[41] Ouédraogo AL, Schneider P, De Kruijf M, Nébié I, Verhave JP, Cuzin-Ouattara N, et al. Age-dependent distribution of *Plasmodium falciparum* gametocytes quantified by Pfs25 real-time QT-NASBA in a cross-sectional study in Burkina Faso. Am J Trop Med Hyg. 2007;76:626–30. 17426160

[42] Balogun ST, Sandabe UK, Bdliya DN, Adedeji WA, Okon KO, Fehintola FA. Asymptomatic falciparum malaria and genetic polymorphisms of Pfcrt K76T and Pfmdr1 N86Y among almajirai in northeast Nigeria. J Infect Dev Ctries. 2016;10:290–7. 10.3855/jidc.6853 27031449

[43] Santolamazza F, Avellino P, Siciliano G, Yao FA, Lombardo F, Ouédraogo JB, et al. Detection of *Plasmodium falciparum* male and female gametocytes and determination of parasite sex ratio in human endemic populations by novel, cheap and robust RTq PCR assays. Malar J. 2017;16:468. 29149898 10.1186/s12936-017-2118-z PMC5693539

[44] Tadesse FG, Meerstein-Kessel L, Gonçalves BP, Drakeley C, Ranford-Cartwright L, Bousema T. Gametocyte sex ratio: the key to understanding *Plasmodium falciparum* transmission? Trends Parasitol. 2019;35:226–38. 30594415 10.1016/j.pt.2018.12.001 PMC6396025

[45] Graves P, Wirtz R, Carter R, Burkot T, Looker M, Targett G. Naturally occurring antibodies to an epitope on *Plasmodium falciparum* gametes detected by monoclonal antibody-based competitive enzyme-linked immunosorbent assay. Infect Immun. 1988;56:2818–21. 2459062 10.1128/iai.56.11.2818-2821.1988 PMC259655

