# Supplementary material for: Plasmodium falciparum gametocyte burden in a Tanzanian heterogeneous transmission setting
Source: Malar J. 2025 Feb 21;24:54. doi: 10.1186/s12936-025-05270-4 (PMC11846475; doi:10.1186/s12936-025-05270-4)
Supplement: Supplementary file 1 — Additional file 1. Primers and probes used for gametocyte detection and quantification by RT‒qPCR. [file 12936_2025_5270_MOESM1_ESM.docx]

Additional file 1: Primers and probes used for gametocyte detection and quantification by RT-qPCR

| Gene ID | Gene name | Oligo sequence | Oligo modification [5’-3’] | Concentration | Adapted from |
| --- | --- | --- | --- | --- | --- |
|  |  | Gametocyte detection and quantification |  | 5x Oligo Mix |  |
| *PF3D7_0903800* | *CCp4 fwd* | CAC ATG AAT ATG AGA ATA AAA TTG | - | 4.5 µM | *Meerstein‑Kessel* et al, 2018 |
|  | *CCp4 rev* | TAG GCG AAC ATG TGG AAA G | - | 4.5 µM | *Meerstein‑Kessel* et al, 2018 |
|  | CCp4 probe | AGC AAC AAC GGT ATG TGC CTT AAA ACG | TexasRed-BHQ2 | 0.625 µM | *Meerstein‑Kessel* et al, 2018 |
| *PF3D7_1469900* | *PfMGET fwd* | CGG TCC AAA TAT AAA ATC CTG | - | 1.125 µM | *Meerstein‑Kessel* et al, 2018 |
|  | *PfMGET rev* | TGT GTA ACG TAT GAT TCA TTT TC | - | 1.125 µM | *Meerstein‑Kessel* et al, 2018 |
|  | *PfMGET probe* | CAG CTC CAG CAT TAA AAA CAC | FAM – BHQ2 | 0.625 µM | *Meerstein‑Kessel* et al, 2018 |
