# Supplementary material for: Plasmodium falciparum gametocyte burden in a Tanzanian heterogeneous transmission setting
Source: Malar J. 2025 Feb 21;24:54. doi: 10.1186/s12936-025-05270-4 (PMC11846475; doi:10.1186/s12936-025-05270-4)
Supplement: Supplementary file 2 — Additional file 2. RT‒qPCR reaction efficiency for PfMGET and CCp4 markers used for the detection and quantification of male and female gametocytes, respectively, in the malaria survey participants. [file 12936_2025_5270_MOESM2_ESM.docx]

Additional file 2 : RT-qPCR reaction efficiency for PfMGET and CCp4 markers used for the detection and quantification of male and female gametocytes, respectively, in the malaria survey participants
