# Supplementary material for: Plasmodium falciparum gametocyte burden in a Tanzanian heterogeneous transmission setting
Source: Malar J. 2025 Feb 21;24:54. doi: 10.1186/s12936-025-05270-4 (PMC11846475; doi:10.1186/s12936-025-05270-4)
Supplement: Supplementary file 3 — Additional file 3. Malaria parasite positivity RDT and microscopy. [file 12936_2025_5270_MOESM3_ESM.docx]

Additional file 3: Malaria parasite positivity by mRDT and Microscopy, N = 467

| Demographic variables | Malaria positivity by mRDT | | Asexual positivity by Microscopy | | | | Gametocyte positivity by Microscopy | | |
| --- | --- | --- | --- | --- | --- | --- | --- | --- | --- |
|  |  |  |  |  |  |  |  |  |  |
|  | n^r^ | %  (95% CI) | | n^a^ | %^a^  (95% C I) | mean count/µl (95% CI) | n^s^ | %^s^  (95% CI) | mean count/µl  (95% CI) |
| Overall | 77 | 16.5  (13.4 – 20.1) | | 73 | 15.6  (12.6 – 19.2) | 1170.0  (711.2 – 1924.8) | 17 | 3.6  (2.3 – 5.8) | 38.7  (27.4 – 54.5) |
| Age-group | | | | | | | | | |
| Children | 41 | 21.4  (16.1 – 27.7) | | 43 | 22.4  (17.0 – 28.9) | 1374.6  (664.7 – 2842.8) | 8 | 4.2  (2.1 – 8.1) | 35.1  (21.9 – 56.3) |
| Adolescent | 17 | 26.2  (16.9 – 38.1) | | 13 | 20.0  (12.0 – 31.5) | 662.4  (402.9 – 1089.1) | 3 | 4.6  (1.5 – 13.4) | 40.3  (14.9 – 109.0) |
| Adult | 19 | 9.0  (5.8 – 13.8) | | 17 | 8.0  (5.1 – 12.6) | 1200.5  (383.6 – 3757.2) | 6 | 2.9  (1.3 – 6.2) | 43.0  (16.0 – 115.5) |
| Sex | | | | | | | | | |
| Female | 35 | 13.9  (10.1 – 18.7) | | 34 | 13.5  (9.8 – 18.3) | 1563.3  (611.7 – 3995.7) | 5 | 2.0  (0.8 – 4.7) | 42.1  (17.5 – 101.3) |
| Male | 42 | 19.5  (14.8 – 25.4) | | 39 | 18.1  (13.5 – 23.9) | 914.5  (557.9 – 1498.9) | 12 | 5.6  (3.2 – 9.6) | 37.3  (24.2 – 57.4) |

N = total number of participants tested

n^r^ = number of participants who tested positive by mRDT

n^a^ = number of participants who tested positive for asexual parasites

n^s^ = number of participants who tested positive for gametocytes

%^a^ = n^a^/N*100

%^s^ = n^s^/N*100
